# Phylogenetic Analysis of Hepatitis B Virus Genotypes Circulating in Different Risk Groups of Panama, Evidence of the Introduction of Genotype A2 in the Country

**DOI:** 10.1371/journal.pone.0134850

**Published:** 2015-07-31

**Authors:** Alexander A. Martínez, Yamitzel Zaldívar, Griselda Arteaga, Zoila de Castillo, Alma Ortiz, Yaxelis Mendoza, Omar Castillero, Juan A. Castillo, Juan Cristina, Juan M. Pascale

**Affiliations:** 1 Department of Genomics and Proteomics, Gorgas Memorial Institute for Health Studies, Panama City, Panama, Panama; 2 Department of Biotechnology, Acharya Nagarjuna University, Guntur City, Andhra Pradesh, India; 3 INDICASAT-AIP, Clayton, City of Knowledge, Panama; 4 Nucleic Acid Test Unit, Complejo Hospitalario Dr. Arnulfo Arias Madrid, Caja de Seguro Social, Panama City, Panama; 5 Department of Microbiology, School of Medicine, University of Panama, Panama City, Panama, Panama; 6 Laboratorio de Virología Molecular, Centro de Investigaciones Nucleares, Facultad de Ciencias, Universidad de la República, Igua, Montevideo, Uruguay; CRCL-INSERM, FRANCE

## Abstract

The Hepatitis B Virus (HBV) can cause acute or chronic infection it is also associated with the development of liver cancer, thousands of new infections occur on a yearly basis, and many of these cases are located in certain areas of the Caribbean and Latin America. In these areas, the HBV prevalence is still high which makes this virus a serious public health concern to the entire region. Studies performed in Panama suggest a complex pattern in the distribution of HBV among the country’s different risk groups. We use phylogenetic analysis in order to determine which HBV genotypes were circulating in these specific groups; for this we used a fragment of the PreS2/2 region of the HBV genome. Subsequently whole HBV genome sequences were used for Bayesian analysis of phylodynamics and phylogeography. Two main genotypes were found: genotype A (54.5%) and genotype F (45.5%). There was a difference in the distribution of genotypes according to risk groups: 72.9% of high risk groups were associated to genotype A, and 55.0% of samples of genotype F were associated to the low risk group (p<0.002). The Bayesian analysis of phylogeny-traits association revealed a statistically significant geographical association (p<0.0001) with both genotypes and different regions of the country. The Bayesian time of most recent common ancestor analysis (tMRCA) revealed a recent tMRCA for genotype A2 circulating in Panama (1997, 95% HPD: 1986—2005), when it is compared with Panamanian genotype F1c sequences (1930, 95% HPD: 1810 – 2005). These results suggest a possible change in the distribution of HBV genotypes in Panama and Latin America as a whole. They also serve to encourage the implementation of vaccination programs in high-risk groups, in order to prevent an increase in the number of new HBV cases in Latin America and worldwide.

## Introduction

Hepatitis B Virus (HBV) is a major cause of serious liver disease [1]. The virus can cause acute or chronic infection and is commonly associated with the development of liver cancer. Although an effective vaccine for HBV was approved over a decade ago, there are still thousands of new infections occurring every year, many of these cases are located in certain areas of Asia, Africa and the Caribbean where the HBV prevalence is high, making this virus a serious public health concern [2].

In Panama, HBV prevalence rates vary according to the population being studied. Commercial sex workers (CSW) show a low prevalence (0.9%) [3]; men that have sex with men (MSM) have an intermediate prevalence (3.4%) [4], while blood donors have the lowest prevalence (0.03%) [5]. These differences in HBV prevalence between the different groups has also been observed in other countries [6–16]. This could serve as an indication that some individuals are at a higher risk for acquiring the infection [17], resulting in a particular pattern of distribution among groups, or an expansion of best fitted virus strains among different HBV genotypes circulating in the country [18].

HBV is classified in genotypes A to I according to divergences greater than 8% in its genome [19]. Several studies suggest that the distribution of the HBV genotypes is associated with worldwide human migration [20–22]. Additionally, recent studies have demonstrated that the local circulation of HBV genotypes also have components that are related to ethnicity and risk behaviors [9,23,24]. Bayesian studies have revealed that genotype F has a long history of local circulation within Latin America [25–28], however, recent surveys have reported the circulation of genotypes A, B, C, D [29,30], and the presence of recombinant forms of genotype A and F in the region [31] across the continent [15,32–38]. These findings suggest new dynamics in the circulation of HBV genotypes and the introduction of new genotypes in Central and South America.

To explore the hypothesis that the circulation of HBV genotypes in Panama are not only associated with geographic location, but also with population behaviors, we evaluated the prevalence of different HBV genotypes from three cohorts of different Panamanian subjects, stratified by geographic origin and transmission risk factor. The purpose of this study was to evaluate the possible association of HBV genotypes to explore the probable origin of the genotypes circulating in the country and with different possible risk groups.

## Materials and Methods

### Ethics statement

The study was evaluated and approved by the Gorgas Memorial Institute Review Board. All subjects signed informed written consent and were in agreement with the use of their samples for additional studies.

### Sampling

We evaluated positive samples of HBsAg from three different risk groups: commercial sex workers (CSW) (n = 6), men that have sex with men (MSM) (n = 28), and HIV positive subjects (n = 34). All these samples were obtained during an epidemiologic study of HIV and STD in Panama, which was carried out from 2010 to 2013. HBV-DNA positive samples from blood donors (n = 88) (a low risk group) were also analyzed. Samples from blood donors were obtained from a previously described study [27].

### Molecular analysis

DNA was extracted from plasma (300μL) using Qiamp Reagent according to the manufactured procedure (Qiagen CA). The HBV viral load was quantified by using a previously described method [39]. Samples with more than 2,000 viral copies/ml were subjected to a nested PCR to amplify a portion (879 bp) of the HBV genome (nucleotides 221 to 1100 relative to reference AB036910 strain) which allowed us to differentiate HBV at the sub-genotype level [27].

The HBV genotype was first determined by using a web-based program [40] and confirmed by phylogenetic analysis. A dataset with the Panamanian HBV sequences and HBV reference sequences, obtained from Genbank, were aligned by using Muscle v3.8.31 [41]. Maximum Likelihood (ML) phylogenetic trees were inferred under GTR+I+Γ nucleotide substitution model which was selected by using the jModeltest program [42]. The ML tree was reconstructed with the PhyML program, for which we used an online web server [43]. The reliability of the obtained topology was estimated by using an approximate likelihood-ratio test (αLTR) based on the Shimodaira-Hasegawa-like procedure [44]. The obtained tree was visualized in FigTree v1.4.2 (available at http://tree.bio.ed.ac.uk/software).

Nucleotide changes in PreS2-S, HBsAg, Basal Core Promoter region (BPC), preCore and X gene regions (X/preC) and amino acid substitutions in polymerase, were determined by evaluating the Panamanian nucleotide sequences or the translated sequence against reference genotypes using MEGA 5.2 [45].

Amplification of the full-length genome of Panamanian sequences were carried out using primers P1 and P2 published elsewhere [46]. Samples with low viral load copy (<10,000 copies/ml) were amplified through the use of a nested PCR protocol [26].

### Correlation of the HBV phylogeny with geographical and risks behavioral traits

In order to test for statistical significance in the observed phylogeny-trait association of high risk groups and geographic location in Panama, a Bayesian Tip-Significant test (BaTS) [47] was applied. The dataset consisted of 88 Panamanian sequences, 879 bp long. A set of 200 trees, representing the phylogenetic structure of the sequences of the HBV strains circulating in the country, was obtained using BEAST v1.8 [48], the parameters of the analysis were the following: GTR+I+Γ substitution model, with 50 million MCMC computed states, and the sampling of trees every of about 200,000 generations, after 20% of burn-in. Each sequence was labeled with its corresponding risk group and geographic location. The Association index (AI), the parsimony score statistics (PS), and the monophyletic clade size statistics (MC) (from the 200 trees) were calculated with BaTS and the null distributions of each statistic were obtained with 1000 random permutations.

### Bayesian analysis of population history and phylogeography analysis of the genotypes F and A

Whole genome HBV sequences obtained at different times were used to estimate the evolutionary rates (*μ*, nucleotide substitutions per site per year, s/s/y), and the time to the most common recent ancestor (*T*_mrca,_ years) of the Panamanian sequences with a dataset from the genotype F (n = 94) classified by sub-genotypes: F1a (n = 3), F1a-pan (n = 2), F1b (n = 24), F1c-pan (n = 7), F2 (n = 9), F3 (n = 32), F4 (n = 15), F5-pan (n = 2) and a dataset from the genotype A (n = 271) also classify by sub-genotype as follow: A1 (n = 71), A2 (n = 161), A3 (n = 37), A4 (n = 2). To infer the tMRCA of these sub-genotypes we used time-stamped data and an intermediate rate of substitution (1.0 x 10^−5^ s/s/y) as was suggested by Torres et. al [26]. To test the hypothesis that the Panamanian sequences have a similar origin, we studied the spatial diffusion of the Panamanian samples obtained in this study evaluating the phylogeography of the complete genome sequences of genotype F, genotype A and sub-genotype A2. For the genotype F the database was based on sequences previously published [26], and sequences of Panamanian origin. For the genotype A and A2, sequences were retrieved in May 2014 from Genbank, the accession numbers, collection dates, and location data of the used sequences are in S1 File. The sequences were then evaluated with RDP4 software to discard recombinants [49], additionally, sequences with a common origin (i.e.: nosocomial outbreaks) were discarded. The final datasets consisted of 94 sequences for genotype F, 116 sequences for genotype A. Both datasets had geographic location information and sampled dates, and 139 sequences for sub-genotype A2 with just geographic location information. All alignments are available upon request. The evolutionary rates, the tMRCA and the spatial diffusion were calculated simultaneously in BEAST v1.8. We used a partitioned model of the whole genome of HBV, with different substitutions models according to the region of the HBV genome [28]. A relaxed uncorrelated lognormal model was used as molecular clock, a Bayesian skyline plot as a tree model [50] and a reversible discrete diffusion model was used [51] for the analysis of phylogeography. The MCMC chains were run for 200 million of generations and the convergence (ESS>200) was evaluated in TRACER v1.6. The obtained trees were summarized with Tree annotator v1.8 and the Maximum credibility tree was visualized in FigTree v.1.42 (available at http://tree.bio.ed.ac.uk/software).

The sequences used in this study were deposited in Genbank under the accession numbers KP718063 to KP718113

## Results

### The distribution of HBV genotypes in Panama is significantly associated with the geographic location

**Fig 1:**
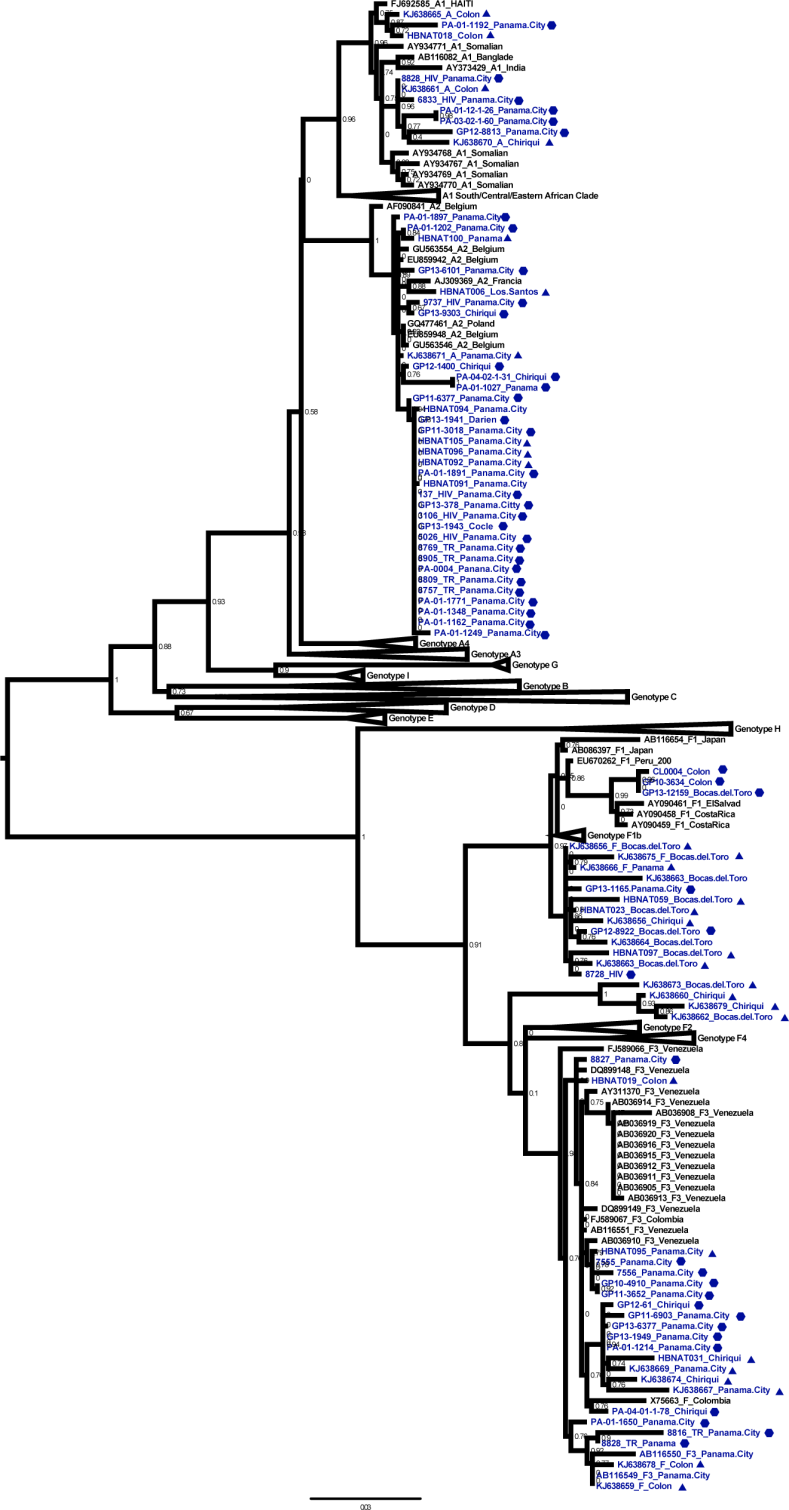
Maximum Likelihood tree of HBV Panamanian sequences. The tree is based on HBV partial PreS2-S region (~820pb), using reference sequences of each genotype (A-I) (n = 280) and sequences of Panamanian origin (n = 88) which are colored in blue. The place of sample collection is indicated in the taxa name, the symbols represent the behavioral risk groups to which the sample belongs: bold triangle: low risk, bold hexagon: high-risk group (bold circle). Clades in which there are not Panamanian samples are collapsed to simplify, node numbers correspond to αLTR values higher than 0.75.

**Fig 2:**
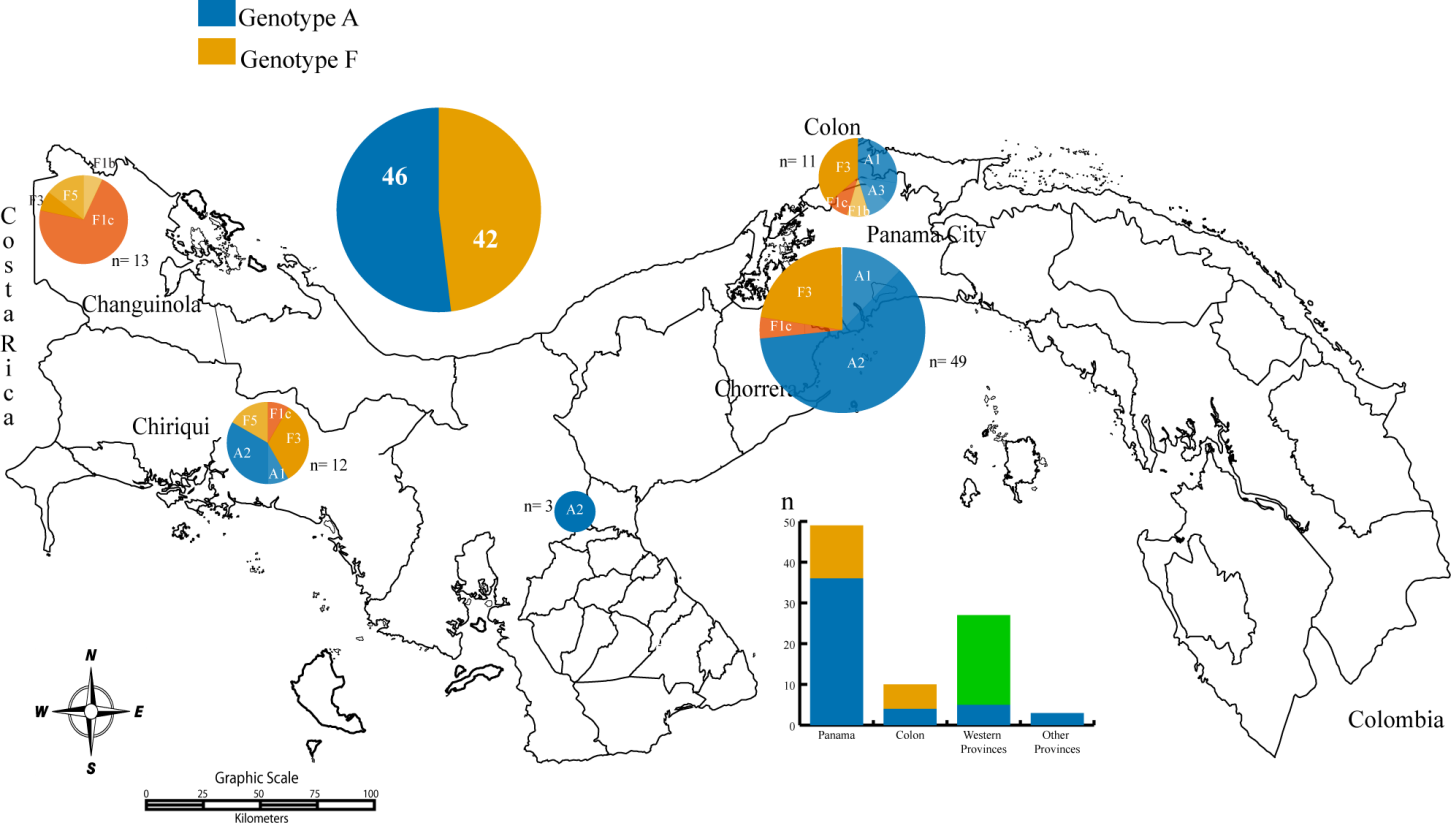
Map of Panama indicating the origin of the HBV sequences analyzed in this study. The number of sequences according to the genotype is indicated in the top left pie chart, the middle bar plot indicates the number of genotypes according to geographic area. Pie charts within the map are sized by the proportion of sub-genotype found in the corresponding region.

In this study we evaluated the HBV genotypes that are circulating in four different Panamanian populations of HBV positive subjects. We grouped these populations into two risk levels: low risk (blood donors) and high risk (CSW, MSM, HIV positive subjects). A total of 156 HBsAg positive subjects were included in the study and 88 partial PreS2-S sequences were obtained from them. Genotype A was the most prevalent and detected in 48 (54.5%) subjects, followed by genotype F in 40 (45.5%) participants (Fig 1). When we grouped the sequences according to the risk of acquiring the infection, we obtained the following results: the low risk group showed a significant association with genotype F (n = 22, 55%), however, high risk groups showed a significant association with genotype A (n = 35, 72.9%) (p<0.002) Table 1. When the samples were analyzed according to geographical location, associations were noted for the genotype F in Western provinces of Panama (n = 21, 81%), whereas genotype A was concentrated in the central areas of the country (n = 37, 75.5%). Table 1 (Fig 2).

**Table 1:** Genotype distribution of the HBV sequences evaluated according to risk behavior group, geographic location and the mutations found in BCP, X/PreC and Polymerase.

	Genotype			
	Total (n = 88)*	A	F	p value^&^
**Risk group**				
Blood donors	35 (39.8)	13 (14.8)	22 (25.0)	0.002
CSW°	4 (4.5)	3 (3.4)	1 (1.1)	
MSM°°	22 (25.0)	17 (19.3)	5 (5.7)	
HIV Positive	27 (30.6)	15 (17.0)	12 (13.6)	
Total	88	48 (54.5)	40 (45.5)	
**Geographic location**				
Panama City	49 (55.6)	37 (42.0)	12 (13.6)	0.0001
Colon	10 (11.3)	4 (4.5)	6 (6.8)	
Western countries^$^	26 (29.7)	5 (5.7)	21 (24.0)	
Other countries^$$^	3 (3.4)	3 (3.4)	0	
**Mutations Evaluated**				
BCP region**(n = 44)		0	4 (9.1)	
X/PreC *** (n = 44)		1 (2.3)	8 (18.2)	
Polymerase ****(n = 88)		3 (3.4)	1 (1.1)	

Data are: Number (%)

* This number represent the total of samples successfully amplified.

^&^Chiriqui, Bocas del toro, Ngobe Bugle.

^$$^Los Santos, Cocle.

°Commercial sex workers

°°Men that have sex with Men.

^&^Exact-Fisher test statistic.

**Double mutation 1762T/1764.

*** Any combination of mutations: 1613A, 1653T, 1846T, 1896, 1899A as suggested by Park et al (49).

****All the subjects with resistance mutation were HIV positive.

During sub-genotype analysis the tree topology formed by the sub-genotype A2 and sub-genotype F3 sequences showed a probable association with high-risk traits. In order to evaluate trait diffusion among high-risk groups and geographic location, a Bayesian association-tips significant test (BaTS) was implemented with all the Panamanian sequences generated by this study (n = 88). The analysis showed a significant spatial phylogeography structure in the Panamanian sequences (p< 0.005), with significant clustering of genotype A in Panama City and genotype F in the western region of the country in Changuinola Table 2. The analysis of association with risk to acquire infection showed no statistically significant clustering among risk groups, therefore, the results do not discard the possibility that the observed high-risk association with genotype A could have been a result of coincidence (p >0.05) Table 2.

**Table 2:** Bayesian phylogeny-traits association results, according to risk behavior group and geographic location.

	Estimated mean (95% HPD CI)	p-value
**Risk Group**		
AI	6.12 (5.6, 7.33)	0.005
PS	40.94 (37.6, 43.89	0.006
MC (HIV positive subjects)	2.43 (1.96, 3.18)	0.12
MC (MSM group)	2.20 (1.67, 3.02)	0.74
MC (Blood donors)	2.97 (2.33, 4.14)	0.08
**Geographic location**		
AI	6.18 (5.46, 6.86)	<0.000
PS	35.4 (33.50, 36.93)	<0.000
MC (Panama)	4.22 (3.24, 5.80)	0.003
MC (Colon)	1.37 (1.03, 2.0)	0.070
MC (Changuinola)	1.55 (1.05, 2.07)	0.006
MC (Chiriqui)	1.44 (1.05, 2.01)	1

Abbreviations: Association index (AI), parsimony score (PS), and monophyletic clade (MC), Highest posterior density (HPD), confidence interval (CI). For the MC statistic the corresponding trait is indicated between parentheses, for p value calculation the desired level of significance was ≤0.05.

### Recent population history from Panamanian samples of HBV genotype A

To estimate the rates of nucleotide substitutions and the tMRCA of genotype A and F, we used a Bayesian coalescence approach under an uncorrelated lognormal relaxed molecular clock with a Bayesian skyline tree model. This model showed the best descriptions of the evolutionary history of HBV [5,26]. For genotype A, the mean rate of substitutions 4.39x10^-04^ subs/site/year (95% HPD, 2.2 x 10^−4^–7.0 x 10^−4^) were higher than the mean rates for the genotype F, 1.20 x 10^−04^ subs/site/year (95% HPD, 7.0 x 10^−6^, 2.0 x 10^−4^) with no overlap among HPD intervals Table 3. The analysis of tMRCA of genotype F confirms previous estimates of tMRCA for each sub-genotype (Table 3), but when Panamanian samples of sub-genotype F1a were evaluated (F1a pan in Table 3), we observed younger tMRCA compared with others genotype reported in America: F1a, F2, F3, F4, and even with sub-genotype F1c and F5 that were also reported in Panama.

**Table 3:** Estimated of tMRCA and substitution rate for Genotype A and F, with the HBV Whole genome of the Panamanian samples.

		Whole Genome				
		(Partitioned model)				
		Time-stamped data			(Fixed subtitution rate 1.0 x 10^−5^ subs/site/year)	
Genotype	Group	tMRCA (years)	95% HPD interval (years)	substitution rate (subs/site/year) (95% HPD interval)	tMRCA (years)	95% HPD interval (years)
**A**	A	212	92–359		5295	3433–7728
	A1	144	70–224		4811	3054–6737
	A1 pan	3	1–6		70	6–138
	A2	46	28–68	4.39 x 10^−4^	2367	1488–3479
	A2 pan	17	7–27	(2.2 x 10^−4^ -	1133	942–1335
	A3	87	45–142	7.0 x 10^−4^)	4045	2451–6376
	A4	24	15–35		768	171–1121
**F**	F	410	182–704		5907	3263–9008
	F1a	82	37–163		580	274–914
	F1a pan	9	3–20		127	11–301
	F1b	114	43–245		857	525–1232
	F1c pan	104	32–233		867	521–1226
	F2	185	63–392	1.20 x 10^−4^	1784	938–2735
	F3	117	47–242	(7.0 x 10^−6^ -	1036	675–1480
	F3 pan	118	47–246	2.0 x 10^−4^)	1058	681–1512
	F4	120	44–255		1065	578–1732
	F5 pan	92	11–211		1032	349–1954

Abbreviations: tMRCA, mean time to the most recent common ancestor; 95% HPD, 95% highest posterior density; subs/site/year, substitutions per site year.

**Fig 3:**
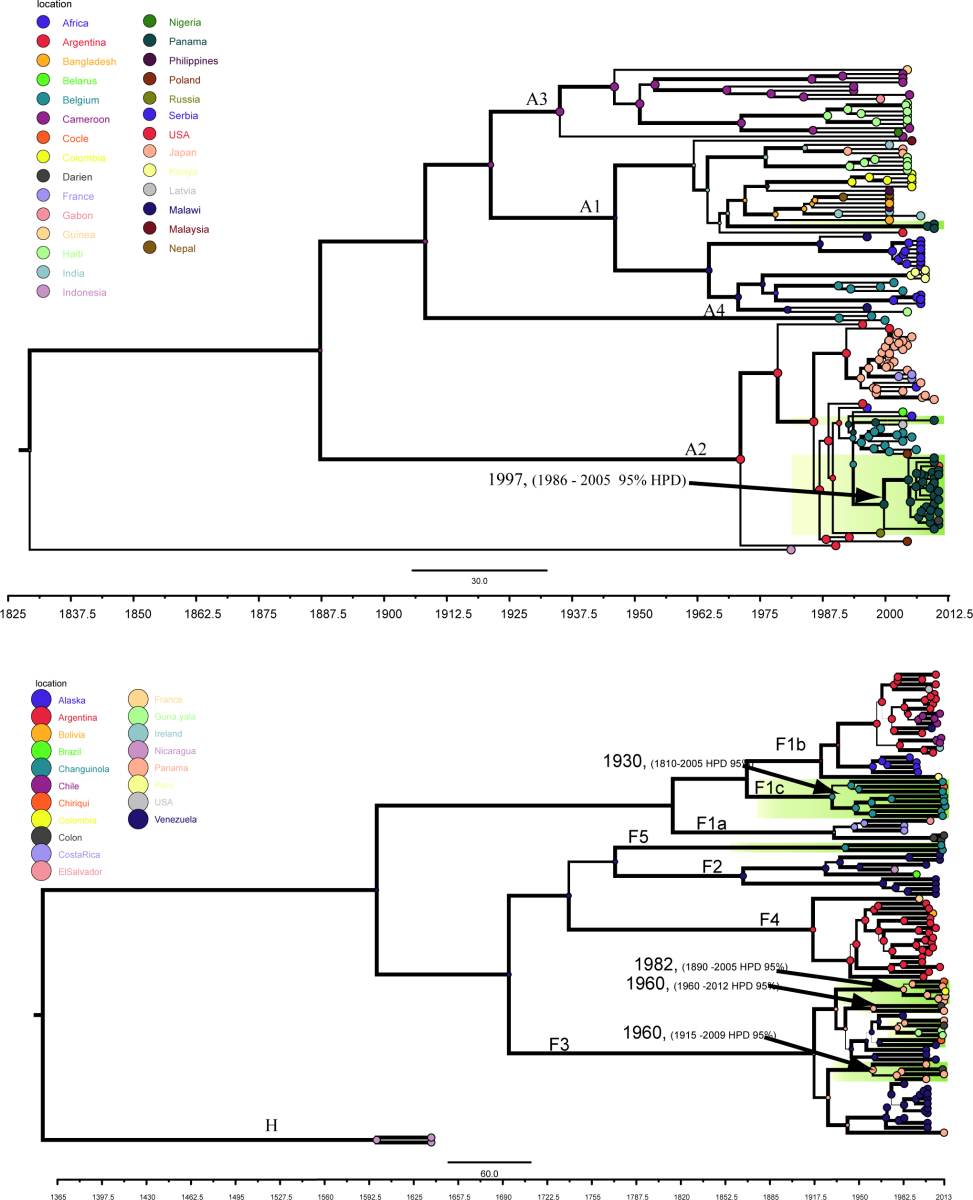
MCC trees showing the time resolved phylogenies of the Panamanian samples. The tree was draw using the whole genome sequences of the Panamanian samples and sequences published elsewhere for genotypes A (a) and F (b). The date of MRCA is shown in the node of relevant sub-genotypes and nodes formed by Panamanian samples (shadowed). The most probable location states of each node are indicated by colored circles, the circles are sized by state posterior probability. The thickness of the Branch lines indicates the Bayesian posterior probabilities of each clade branch with the lower value set as 1 and the higher as 3 in Figtree. *Indicates the monophyletic clade analyzed in the tMRCA analysis.

The tMRCA analysis of the sub-genotype A2 showed that the MRCA of this sub-genotype dated around 1997 (1986–2005) (Fig 3A). This result contrasts with the older circulation dates for the MRCA of the Panamanian genotype F: 1930 (1810–2005) for the genotype F1c, and 1960 (1915–2009) for the genotype F3 (Fig 3B).

### Different geographic origin of genotype F and genotype A2 in Panama

**Fig 4:**
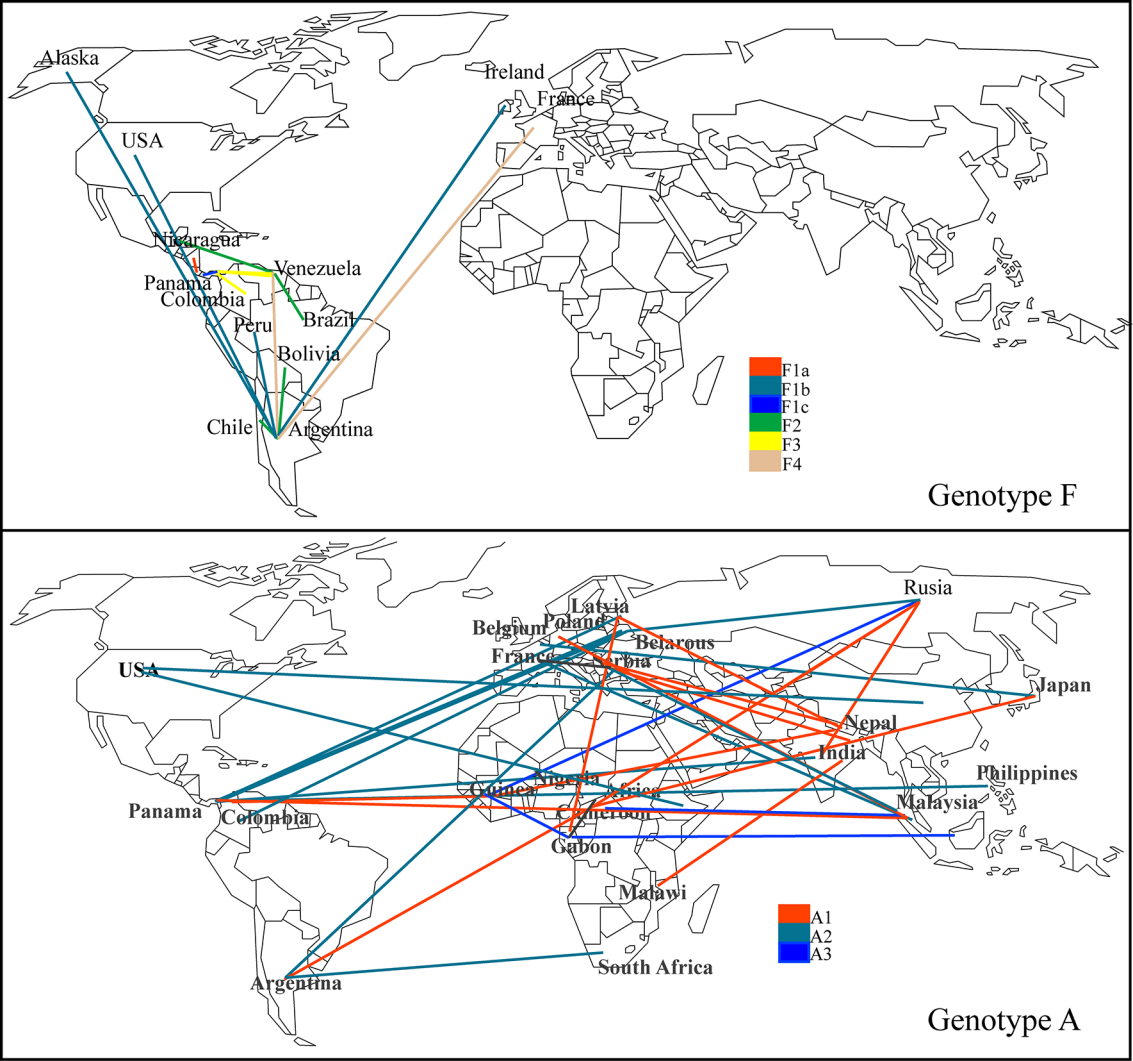
Spatial dissemination of HBV genotypes across the world. Lines between countries represent sequences in which transition location occurs, and were supported by a significant non-zero rate (Bayes Factor >3). Map (a) is for genotype F (a), and map (b) for genotype A. Lines were colored according to the sub-genotype involved in the migration.

The most probable circulation place for Genotype F is the American continent (Fig 4B). Particularly in Panama, there is circulation of genotypes F3, F1a, F1c and F5. Phylo-geographic results for Sub-genotype F3 suggest a circulation of this sub-genotype among Venezuela, Panama, and Colombia (Fig 4A), with a similar tMRCA for all these F3 sequences (Table 3). However, the sub-genotype F1c and sub-genotype F5 are exclusively located in Panama. The sub-genotype F1c has a more recent tMRCA compared with F5, but with a 95% HPD overlap between its tMRCA confidence intervals.

When we analyzed the geographical origin of genotype A, in Panama this genotype seems to have a different origin from the one of genotype F (Fig 3A and 3B). The sub-genotype A1 found in Panama have a significant association with strains circulating in Central Africa, instead the sub-genotype A2 have relation with strains circulating in Europe and Russia (Fig 4B).

## Discussion

The HBV genotypes circulation in Panama depends mainly on the geographic location of the samples. The association of genotypes with risk groups was not confirmed by Bayesian phylogenetic analysis. Although studies performed in countries such as Japan, United States and Belgium suggested an increased prevalence of genotype A2 in acute infections and in high-risk groups [14,52–54], our data did not support statistically significant monophyletic clades association, with HBV genotype circulation in MSM or other behavioral risk traits. When we compare the genotype distribution in the present study with a previous study that included only Panamanian blood donors (low risk group) [5], a difference in prevalence of genotype F and A was clearly observed. The differences in genotype distribution suggest that there is an association between risk behavior and HBV genotypes in Panama.

Sub-genotype analysis showed differences in the HBV genotype circulation in the country; with genotypes A2 and F3 located in Panama City while the recently described genotypes F1c and F5 in the western areas of Panama. These results are of extreme importance, because based on previous studies Panamanian samples of genotypes F had a higher proportion of mutations related with the development of hepatocellular carcinoma in the BCP and X/PreC regions [27]. Therefore, subjects infected in the western areas of Panama had a greater chance of being infected with a virus variant that basically has a much higher probability of inducing the development of hepatocellular carcinoma [55,56].

In the present study we also found that most of the samples from HIV positive individuals with antiretroviral resistance mutations were infected with genotype A, a situation that was observed in countries such as Japan [14] and The Netherlands [57]. This suggests that HIV positive subjects who are co-infected with HBV in both Panama City and elsewhere must be evaluated for resistant mutations before initiating treatment.

Previous studies demonstrated that genotype F is in fact the oldest genotype circulating in the Americas [26,27]. In a previous study, we reported a tMRCA for genotype F1c (1911, HPD95% 1636–1975) [5] which is in agreement with the historical period of high population density during the construction of the Panama Canal [58] In the present study, we observed a recent tMRCA for genotype A2, and for sub-genotype F1a circulating in Panama. These results evidence the co-circulation of different HBV genotypes in Panama and suggest a possible recent introduction of genotype A2 and F1a in the country. In addition, the higher substitution rates observed in genotype A samples, could be the result of an increased number of susceptible hosts, as is the case of Panamanian MSM subjects, where the risk of acquired sexually transmitted diseases is increased [4]

The recent introduction of sub-genotype F1a in Panama, suggests the possibility that there is a beginning of dissemination of this HBV sub-genotype from Central America to South America. There is a need to perform studies aimed to evaluate circulation of HBV genotypes circulating in Central America and Mexico to better explain the spatial diffusion of this sub-genotype in the population of this part of the continent.

The phylogeography analysis we are presenting confirms the circulation of genotype F across the American continent, this observation together with the similar tMRCA results for sub-genotypes of genotype F, supports the hypothesis of early spread of genotype F in the continent after its origin from a common ancestor several years ago. Additionally, the extended worldwide migration of genotype A, especially genotype A2 supports a panmixis hypothesis of genotype A2 across the world. Moreover, there is an increasing evidence of a recent expansion of the genotype A2 in different areas of the world. For example, in Japan a steady increase in genotype A2 cases has been observed in HIV positive MSM subjects [14]. However, in the United States, the expansion of the genotype A2 has been observed in acute infections [59]. Therefore, the growing number of cases of genotype A2 in Panama could be part of a global increase in the circulation of the genotype A2. This is particularly important because we found that genotype A has a slightly higher level of nucleotide substitution rates (Table 3) when compared to genotype F. This could lead to a potential increase in the emergence of new variants with effective transmission in susceptible subjects.

## Conclusion

We found that HBV genotype distribution in Panama is changing. There has been a recent increase in the circulation of genotype A in the country, and the emergence of mutations that could change the clinical prognosis of the infection for both genotypes A and F. Implementation of vaccination programs in high risk groups should be a priority in the region in order to avoid further increases in the number of new HBV cases. This could also reduce the potential increase of genotype A distribution in Latin America and the world.

## Supporting Information

S1 File Data of the sequences analyzed*. *Panamanian sequences generated in this study are shown in bold. n/i: No information).
(PDF)
