# Supplementary material for: Phylogenetic Analysis of Hepatitis B Virus Genotypes Circulating in Different Risk Groups of Panama, Evidence of the Introduction of Genotype A2 in the Country
Source: PLoS One. 2015 Jul 31;10(7):e0134850. doi: 10.1371/journal.pone.0134850 (PMC4521924; doi:10.1371/journal.pone.0134850)
Supplement: S1 File — *Panamanian sequences generated in this study are shown in bold. n/i: No information). (PDF) [file pone.0134850.s001.pdf]

S1 File **Data of the sequences analyzed***

**Accesion number or sample name**	**Collection date**	**Country of collection**	**Genotype**
AB076678	1997	Malawi	A1
AB076679	1997	Malawi	A1
AB116082	2002	Bangladesh	A1
AB116083	2002	Bangladesh	A1
AB116084	2002	Bangladesh	A1
AB116085	2002	Bangladesh	A1
AB116086	2002	India	A1
AB116087	2002	India	A1
AB116088	2002	Nepal	A1
AB116089	2002	Nepal	A1
AB116091	2002	Philippines	A1
AB116092	2002	Philippines	A1
AB116093	2002	Philippines	A1
AB116094	2002	Philippines	A1
AB453986	2005	Japan	A1
AB453987	2005	Japan	A1
AB453988	2005	Japan	A1
AB453989	2005	Japan	A1
EU366129	2005	Argentina	A1
FJ692557	2006	Haiti	A1
FJ692558	2006	Haiti	A1
FJ692559	2006	Haiti	A1
FJ692561	2006	Haiti	A1
FJ692562	2006	Haiti	A1
FJ692563	2006	Haiti	A1
FJ692564	2006	Haiti	A1
FJ692565	2006	Haiti	A1
FJ692566	2006	Haiti	A1
FJ692567	2006	Haiti	A1
FJ692571	2006	Haiti	A1
FJ692572	2006	Haiti	A1
FJ692574	2006	Haiti	A1
FJ692575	2006	Haiti	A1
FJ692576	2006	Haiti	A1
FJ692577	2006	Haiti	A1
FJ692578	2006	Haiti	A1
FJ692579	2006	Haiti	A1
FJ692580	2006	Haiti	A1
FJ692581	2006	Haiti	A1
FJ692582	2006	Haiti	A1
FJ692583	2006	Haiti	A1
FJ692584	2006	Haiti	A1
FJ692585	2006	Haiti	A1
FJ692590	2006	Haiti	A1
FJ692591	2006	Haiti	A1
FJ692592	2006	Haiti	A1
GU563545	2005	Belgium	A1
GU563546	2001	Belgium	A1
GU563547	2003	Belgium	A1
GU563548	2000	Belgium	A1
HM011485	2007	Malaysia	A1
JN182324	2009	South Africa	A1
JN182325	2009	South Africa	A1
JN182328	2009	South Africa	A1
JN182330	2009	South Africa	A1
JN182331	2009	South Africa	A1
JN182332	2009	South Africa	A1
JN182333	2009	South Africa	A1
JQ023660	2007	Colombia	A1
JQ023661	2007	Colombia	A1
JQ023662	2007	Colombia	A1
JQ023663	2007	Colombia	A1
JX154579	2010	Kenya	A1
JX154580	2010	Kenya	A1
JX154581	2010	Kenya	A1
KJ010776	2009	South Africa	A1
KJ010777	2009	South Africa	A1
KJ010778	2009	South Africa	A1

KJ533388	2009	India	A1
**PA-01-12-1-26**	**2012**	**Panama**	**A1**
**PA-03-02-1-60**	**2012**	**Panama**	**A1**
AB116076	2002	USA	A2
AB116077	2002	USA	A2
AB116078	2002	USA	A2
AB116079	2002	Japan	A2
AB116080	2002	Japan	A2
AB116081	2002	Japan	A2
AB126580	2000	Russia	A2
AB205118	2001	Japan	A2
AB453979	2005	Japan	A2
AB453980	2005	Japan	A2
AB453981	2005	Japan	A2
AB453982	2005	Japan	A2
AB453983	2005	Japan	A2
AB453984	2005	Japan	A2
AB453985	2005	Japan	A2
AB480040	2006	Japan	A2
AB549213	2009	Japan	A2
AB697487	2003	Japan	A2
AB697488	2003	Japan	A2
AB697489	2004	Japan	A2
AB697491	2005	Japan	A2
AB697492	2005	Japan	A2
AB697493	2005	Japan	A2
AB697495	2005	Japan	A2
AB697496	2002	Japan	A2
AB697497	2006	Japan	A2
AB697498	2006	Japan	A2
AB697499	2006	Japan	A2
AB697501	2006	Japan	A2
AB697503	2006	Japan	A2
AB697504	2006	Japan	A2
AB697505	2006	Japan	A2
AB697506	2006	Japan	A2
AB697507	2007	Japan	A2
AB697508	2007	Japan	A2
AB697509	2007	Japan	A2
AB697511	2007	Japan	A2
AB697512	2007	Japan	A2
AB775198	2010	Japan	A2
AB775199	2010	Japan	A2
AB775200	2012	Japan	A2
AB775201	2012	Japan	A2
AB778116	2012	Japan	A2
EU414133	2005	Belarus	A2
EU859901	2006	Belgium	A2
EU859906	2007	Belgium	A2
EU859907	2007	Belgium	A2
EU859908	2007	Belgium	A2
EU859910	2007	Belgium	A2
EU859911	2007	Belgium	A2
EU859912	2006	Belgium	A2
EU859913	2006	Belgium	A2
EU859914	2005	Belgium	A2
EU859915	2005	Belgium	A2
EU859916	2005	Belgium	A2
EU859917	2005	Belgium	A2
EU859918	2005	Belgium	A2
EU859919	2005	Belgium	A2
EU859920	2006	Belgium	A2
EU859921	2005	Belgium	A2
EU859922	2005	Belgium	A2
EU859924	2005	Belgium	A2
EU859925	2006	Belgium	A2
EU859926	2005	Belgium	A2
EU859927	2005	Belgium	A2
EU859928	2005	Belgium	A2
EU859938	1999	Belgium	A2
EU859939	1999	Belgium	A2
EU859940	1998	Belgium	A2

EU859941	2000	Belgium	A2
EU859942	2000	Belgium	A2
EU859943	2000	Belgium	A2
EU859944	2000	Belgium	A2
EU859945	2000	Belgium	A2
EU859946	2000	Belgium	A2
EU859947	2001	Belgium	A2
EU859948	2000	Belgium	A2
EU859949	1999	Belgium	A2
EU859951	1999	Belgium	A2
EU859953	1998	Belgium	A2
EU859954	1998	Belgium	A2
EU859955	1998	Belgium	A2
EU859956	1998	Belgium	A2
FJ349224	2000	Belgium	A2
GQ477461	2006	Poland	A2
GQ477462	2006	Poland	A2
GQ477463	2006	Poland	A2
GQ477464	2006	Poland	A2
GQ477465	2006	Poland	A2
GQ477466	2006	Poland	A2
GQ477467	2006	Poland	A2
GQ477468	2006	Poland	A2
GQ477469	2006	Poland	A2
GQ477470	2006	Poland	A2
GQ477472	2006	Poland	A2
GQ477473	2006	Poland	A2
GQ477474	2006	Poland	A2
GQ477475	2006	Poland	A2
GQ477476	2006	Poland	A2
GQ477477	2006	Poland	A2
GQ477478	2006	Poland	A2
GQ477479	2006	Poland	A2
GQ477480	2006	Poland	A2
GQ477482	2006	Poland	A2
GQ477483	2006	Poland	A2
GQ477484	2006	Poland	A2
GQ477486	2006	Poland	A2
GQ477487	2006	Poland	A2
GQ477488	2006	Poland	A2
GQ477490	2006	Poland	A2
GQ477491	2006	Poland	A2
GQ477493	2006	Poland	A2
GQ477494	2006	Poland	A2
GQ477495	2006	Poland	A2
GQ477496	2006	Poland	A2
GQ477497	2006	Poland	A2
GQ477499	2006	Poland	A2
GQ477500	2006	Poland	A2
GQ477501	2006	Poland	A2
GQ477502	2006	Poland	A2
GQ477503	2006	Poland	A2
GQ477504	2006	Poland	A2
GU563550	2004	Belgium	A2
GU563551	2003	Belgium	A2
GU563553	2001	Belgium	A2
GU563554	2008	Belgium	A2
GU563555	2007	Belgium	A2
GU563557	2000	Belgium	A2
GU563558	2005	Belgium	A2
GU563562	2008	Belgium	A2
HE576988	2007	France	A2
HE576989	2007	France	A2
JQ687529	2007	Serbia	A2
JQ687533	2008	Serbia	A2
JQ707299	1996	USA	A2
JX096952	2005	Latvia	A2
JX507080	1997	South Africa	A2
X51970	1990	n/i	A2
X70185	1993	n/i	A2
Z35717	1994	n/i	A2
Z72478	1996	n/i	A2

**137**	**2012**	**Panama**	**A2**
**3106**	**2012**	**Panama**	**A2**
**8757**	**2012**	**Panama**	**A2**
**8809**	**2012**	**Panama**	**A2**
**GP11-3018**	**2011**	**Panama**	**A2**
**GP12-1400**	**2012**	**Ngobe.Bugle**	**A2**
**GP13-1941**	**2013**	**Darien**	**A2**
**GP13-1943**	**2013**	**Cocle**	**A2**
**GP13-378**	**2013**	**Panama**	**A2**
**HBNAT091**	**2013**	**Panama**	**A2**
**HBNAT092**	**2013**	**Panama**	**A2**
**HBNAT094**	**2013**	**Panama**	**A2**
**PA-0004**	**2012**	**Panama**	**A2**
**PA-01-1162**	**2012**	**Panama**	**A2**
**PA-01-1202**	**2012**	**Panama**	**A2**
**PA-01-1249**	**2012**	**Panama**	**A2**
**PA-01-1348**	**2012**	**Panama**	**A2**
**PA-01-1771**	**2012**	**Panama**	**A2**
**PA-01-1891**	**2012**	**Panama**	**A2**
**PA-01-1897**	**2012**	**Panama**	**A2**
AB194949	1994	Cameroon	A3
AB194950	1994	Cameroon	A3
AB194951	1994	Cameroon	A3
AB194952	1994	Cameroon	A3
AM184125	2000	Gabon	A3
FJ692554	2004	Nigeria	A3
FJ692595	2006	Haiti	A3
FJ692596	2006	Haiti	A3
FJ692597	2006	Haiti	A3
FJ692598	2006	Haiti	A3
FJ692599	2006	Haiti	A3
FJ692600	2006	Haiti	A3
FJ692601	2006	Haiti	A3
FJ692602	2006	Haiti	A3
FJ692603	2006	Haiti	A3
FJ692604	2006	Haiti	A3
FJ692605	2006	Haiti	A3
FJ692606	2006	Haiti	A3
FJ692607	2006	Haiti	A3
FJ692608	2006	Haiti	A3
FJ692609	2006	Haiti	A3
FJ692610	2006	Haiti	A3
FJ692611	2006	Haiti	A3
FJ692612	2006	Haiti	A3
FJ692613	2006	Haiti	A3
FN545826	2005	Cameroon	A3
FN545828	2005	Cameroon	A3
FN545829	2005	Cameroon	A3
FN545830	2005	Cameroon	A3
FN545831	2006	Cameroon	A3
FN545832	2006	Cameroon	A3
FN545833	2006	Cameroon	A3
FN545834	2006	Cameroon	A3
FN545835	2006	Cameroon	A3
FN545836	2006	Cameroon	A3
FN545838	2006	Cameroon	A3
GQ161813	2006	Guinea	A3
GQ331047	1998	Belgium	A4
GQ331048	2001	Belgium	A4
AY090458	1983	CostaRica	F1a
AY090459	1983	CostaRica	F1a
AY090461	2002	ElSalvador	F1a
**CL0004**	**2012**	**Panama**	**F1a**
**GP10-3634**	**2010**	**Panama**	**F1a**
AB064316	2001	USA	F1b
AB116552	2003	Venezuela	F1b
AF223963	1996	Argentina	F1b
AF223964	1996	Argentina	F1b
AY179735	2002	Argentina	F1b
DQ823091	2006	Argentina	F1b
DQ823093	2004	Argentina	F1b
DQ823094	2005	Argentina	F1b

DQ823095	2006	Argentina	F1b
EU366118	2004	Argentina	F1b
EU366133	2006	Argentina	F1b
EU670262	2008	Peru	F1b
FJ709462	2005	Chile	F1b
HM585191	2009	Chile	F1b
HM585192	2009	Chile	F1b
HM585195	2010	Chile	F1b
HM585197	2007	Chile	F1b
HQ378247	2009	Ireland	F1b
JN792918	1992	Alaska	F1b
JN792919	1992	Alaska	F1b
JN792920	1987	Alaska	F1b
JN792922	1993	Alaska	F1b
AY090455	1976	Nicaragua	F2
AY311369	1999	Venezuela	F2
DQ899142	2009	Venezuela	F2
DQ899143	2009	Venezuela	F2
DQ899144	2006	Venezuela	F2
DQ899145	2006	Venezuela	F2
DQ899146	2006	Venezuela	F2
DQ899147	2006	Venezuela	F2
X69798	1992	Brazil	F2
AB036905	2000	Venezuela	F3
AB036908	2000	Venezuela	F3
AB036910	1982	Venezuela	F3
AB036911	1983	Venezuela	F3
AB036912	2000	Venezuela	F3
AB036913	1983	Venezuela	F3
AB036914	2000	Venezuela	F3
AB036915	2000	Venezuela	F3
AB036916	2000	Venezuela	F3
AB036919	2000	Venezuela	F3
AB036920	2000	Venezuela	F3
AB116551	1999	Venezuela	F3
AY311370	1999	Venezuela	F3
DQ899148	2006	Venezuela	F3
DQ899149	2006	Venezuela	F3
**7555**	**2012**	**Panama**	F3
**7556**	**2012**	**Panama**	F3
**8816**	**2012**	**Panama**	F3
**8827**	**2012**	**Panama**	F3
AB116549	1999	Panama	F3
AB116550	1999	Panama	F3
**GP10-4910**	**2010**	**Panama**	F3
**GP11-3652**	**2011**	**Panama**	F3
**GP11-6903**	**2011**	**Panama**	F3
**GP12-61**	**2012**	**Panama**	F3
**GP13-1949**	**2013**	**Colombia**	F3
**GP13-6377**	**2013**	**Panama**	F3
**HBNAT018**	**2010**	**Panama**	F3
**HBNAT025**	**2011**	**Panama**	F3
**PA-01-1214**	**2012**	**Panama**	F3
**PA-01-1650**	**2012**	**Panama**	F3
**PA-04-01-1-78**	**2010**	**Panama**	F3
AB166850	2004	Bolivia	F4
AF223962	1996	Argentina	F1b
AF223965	1996	Argentina	F1b
AY179734	1997	Argentina	F4
DQ776247	2004	Argentina	F4
DQ823086	2001	Argentina	F4
DQ823087	2001	Argentina	F4
DQ823088	2001	Argentina	F4
DQ823089	2002	Argentina	F4
DQ823090	2002	Argentina	F4
EF576812	2009	Argentina	F4
EU366116	2004	Argentina	F4
EU366132	1999	Argentina	F4
FJ657519	2001	Argentina	F4
FJ657522	2003	Argentina	F4
FJ657528	2006	Argentina	F4
X75658	1994	France	F4

**HBNAT030**	**2010**	**Panama**	**F5**
**HBNAT040**	**2011**	**Panama**	**F5**
**8728.HIV**	**2012**	**Panama**	**F1c**
**GP12-8922**	**2012**	**Panama**	**F1c**
**HBNAT004**	**2012**	**Panama**	**F1c**
**HBNAT013**	**2010**	**Panama**	**F1c**
**HBNAT047**	**2011**	**Panama**	**F1c**
**HBNAT049**	**2011**	**Panama**	**F1c**
**HBNAT097**	**2013**	**Panama**	**F1c**

*Panamanian sequences generated in this study are shown in bold. n/i : No information.
